# Two is better than one: The effects of strategic cooperation on intra- and inter-brain connectivity by fNIRS

**DOI:** 10.1371/journal.pone.0187652

**Published:** 2017-11-16

**Authors:** Michela Balconi, Laurent Pezard, Jean-Louis Nandrino, Maria Elide Vanutelli

**Affiliations:** 1 Research Unit in Affective and Social Neuroscience, Catholic University of Milan, Milan, Italy; 2 Department of Psychology, Catholic University of Milan, Milan, Italy; 3 Aix-Marseille Université, CNRS UMR 7260, LNIA, Marseille, France; 4 Laboratoire SCALab UMR CNRS 9193, Cognitive and Affective Sciences Laboratory Équipe Dynamique des Émotions et Pathologies (DEeP), University of Lille, Lille, France; Universita degli Studi di Roma La Sapienza, ITALY

## Abstract

Inter-brain synchronization during joint actions is a core question in social neuroscience, and the differential contribution of intra- and inter-brain functional connectivity has yet to be clarified along with the role of psychological variables such as perceived self-efficacy. The cognitive performance and the neural activation underlying the execution of joint actions were recorded by functional Near-Infrared imaging during a synchronicity game. An 8-channel array of optodes was positioned over the frontal and prefrontal regions. During the task, the dyads received reinforcing feedback that was experimentally manipulated to induce adoption of common strategies. Intra- and inter-brain connectivity indices were computed along with an inter-brain/intra-brain connectivity index (ConIndex). Finally, correlation analyses were run to assess the relationship between behavioral and physiological levels. The results showed that the external feedback could modulate participant responses in both behavioral and neural components. After the reinforcing manipulation, there were faster response times and increased inter-brain connectivity, and ConIndex emerged primarily over the dorsolateral prefrontal cortex. Additionally, the presence of significant correlations between response times and inter-brain connectivity revealed that only the “two-players connection” may guarantee an efficient performance. The present study provides a significant contribution to the identification of intra- and inter-brain functional connectivity when social reinforcement is provided.

## Introduction

The natural motivation to form bonds with others as well as to cooperate and act prosocially are fundamental connections between human beings. The so-called “social brain” has become a pivotal focus of interest in neuroscience research to explore the neurophysiological basis of interpersonal behavior [1]. In particular, cooperation can be defined as a social interaction between two or more agents that induces sharing and produces common behavioral actions. Joint actions are directed towards the achievement of specific objectives or common interests that provide an advantage to whomever is involved [2]. A recent study in social neurosciences indicated that comprehension of such complex behaviors can only be obtained by considering the interacting actors as a unique system [3]. In fact, interpersonal interactions occur when somebody’s actions affect the immediate and future outcomes of the other individuals who are involved [4].

Nonetheless, the majority of research within social neuroscience has explored such constructs using single-brain paradigms where a single individual interacts with a computer or with another subject in turn-taking tasks (using off-line measurements) [5–7]. However, such paradigms cannot explain the complexity of such processes in real time. Therefore, starting with a few pioneering studies, an increased number of researchers have shifted towards a “second-person” or “two-person neuroscience” [8] that has led to the hyperscanning paradigm. This approach involves the simultaneous recording of neural activity from multiple participants who are interacting [9] and is based on the underlying principle that during joint actions, people become implicitly coupled [10]. In fact, previous studies have revealed typical patterns of inter-brain synchronization with correlated cortical responses. For example, Cui and colleagues [11] simultaneously recorded the brain activity of two subjects while they played a computer-based game in which they had to cooperate or compete. Inter-brain activity coherence was performed, and the findings highlighted the presence of increased coherence between the two brain signals in the right superior frontal cortices during cooperation but not during competition. Holper and colleagues [12] also analyzed inter-brain connectivity involved in imitation and found increased coherence compared to the control condition. Furthermore, Nozawa and colleagues [13] explored interpersonal neural synchronization during cooperative verbal communication and showed an increased synchronization within the frontopolar cortex. All these studies were conducted with functional Near-Infrared Spectroscopy (fNIRS), which imposes low constraints and is relatively tolerant of head/body motion [14]. fNIRS has thus been proven to be a fundamental tool, since it permits an ecological experimental setting in which participants can behave naturally in a realistic environment.

Nonetheless, to the best of our knowledge, no previous study has directly compared the specific contribution of intra- and inter-brain functional connectivity during cooperation in a hyperscanning paradigm. Thus, a first aim of this study was to compare these two different measures during a cooperative joint task. Functional connectivity can be defined as the temporal correlation between neurophysiological events that are spatially remote. It measures simultaneous coupling between two time series [15]. Furthermore, psychological constructs must be taken into consideration when studying cooperative social performances. For example, when cooperating, the adoption of common strategies is crucial and can be strongly influenced by some psychological processes such as mentalization and self-perception. In fact, perceived self-efficacy during social exchange can influence the motivation to create synergic actions. Previous experiments already investigated the effects of external feedbacks assessing the behavioral performance during cooperative or competitive tasks [16–18]. Results showed that the perception of positive outcomes can induce a superior cognitive performance and is related to the activation of prefrontal areas [17,19]. In particular, the Dorsolateral Prefrontal Cortex (DLPFC) was associated with social exchange, such as perspective taking and theory of mind [20] but also with the suppression of selfish behavior [21] and commitment in significant relationships [22], which are extremely important during cooperation.

Although the role of an external feedback was considered in previous research, its effect on brain (both intra- and inter-) connectivity has yet to be explored. Therefore, the aim of this study was to investigate the relationship between intra and inter-brain functional connectivity and behavioral synchronization during cooperation. A hyperscanning paradigm was applied, and participants were required to synchronize their behavioral performance.

We expected to observe brain and behavioral changes related to the experimental conditions as well as possible correlations. We suppose that based on the social task, the two brains may function in a synergic way (as shown by functional connectivity). The inter-brain connectivity may show the ability of the two brains (and the two subjects) to function in a more similar way in terms of hemodynamic activity. At the same time, the behavioral performance may become more synchronous (similar RTs/ERS performance), since the two subjects should learn to make their performance more similar due to the motivational factors induced by the joint task. Additionally, behavioral and neural levels are supposed to be correlated.

Halfway through the task, participants received feedback on their performance, which was previously manipulated by the experimenter in a manner to induce positive perceived self-efficacy that would influence the construction of joint strategies. We hypothesized that inter-brain functional connectivity could increase after receiving this positive social feedback starting from the assumption that a higher connectivity may be considered a strategic way to induce the brains to “work together” [19]. In contrast, before this feedback, we expected higher intra-brain connectivity as a preliminary intra-personal strategy. Finally, we predicted that both intra- and interconnectivity should involve prefrontal regions, especially the DLPFC, which was demonstrated to be relevant in social and cooperative exchanges [23].

## Methods

### Participants

Thirteen dyads (twenty-six total subjects) were recruited during academic activities (participation in lab activities). They were undergraduate students (M age = 24.08, SD = 1.78, fourteen women). Each couple included same-sex individuals matched for age. They did not meet and were not familiar with each other before the experimental session. The participants were all right-handed, presented normal or corrected-to-normal visual acuity, and provided informed written consent to participate in the study. Exclusion criteria included a history of psychopathology (Beck Depression Inventory, BDI-II, [24]; State-Trait-Anxiety-Inventory, STAI, [25]) for the subjects and their immediate families. No neurological or psychiatric pathologies were revealed, as determined in a preliminary screening phase.

The research was conducted according to the guidelines of the Declaration of Helsinki. It was preliminarily approved by the local ethics committee of the Department of Psychology, Catholic University of Milan. The project was approved in its final version. The data files were stored in the Department repository, as requested by the local ethics committee. The legal responsibility for the data custody is entrusted to the Department Director, who can be contacted to receive a copy of the data. No payment was provided for the subjects' performance, and they provided their consent to participate in the research.

### Procedure

Participants paired in dyads were comfortably seated side-by-side in a moderately darkened room in front of a computer screen located approximately 60 cm from their eyes. They were asked to perform a simple task for sustained selective attention (modified from the original task of Balconi and Pagani [26]). To engage dyads in the task, they were told that some cognitive measures would be used to evaluate their subjective performance since they are usually used to screen future professional career success and teamwork capabilities. The cooperative and joint nature of the game was stressed by telling dyads that their scorings were based on the ability to synchronize their responses in terms of both accuracy (error rates, ERs) and response times (RTs) with the other person. Specifically, they were told that “You have to cooperate with your partner during the task to obtain a good joint performance. During the task, your joined performance will be monitored and specific feedback will be furnished to verify this cooperation.” Thus, the development of a joint cooperative strategy by the dyad was strongly suggested. A dark screen separated the two members of the dyad to prevent visual contact and to avoid any possible effect attributable to nonverbal behavior.

The game included an attentional task that required recognizing target stimuli among non-targets that were based on four different combinations of shape and color, such as triangles vs circles, blue vs green. After memorizing the target stimulus that was displayed on the screen, stimuli were presented one after another. The target stimulus features varied in every experimental block, composed by 25 trials. Each trial was composed of three stimuli. Dyads were instructed to answer all the stimuli by pressing left/right buttons to decide for targets or non-targets. Each stimulus remained on the screen for 500 msec with a 300 msec inter-stimulus interval (ISI). After each trial, subjects received feedback in the form of two up-arrows (high cooperation score); a dash (mean performance); or two down-arrows (low cooperation score).

This feedback period lasted 5000 msec. (including the arrows display for 500 msec.; and a dark screen period lasted 4500 msec, the real post-feedback period used for the successive analysis). Then, an inter-trial interval (ITI) lasted for 5000 msec before the next trial was performed (pre-feedback period).

**Fig 1:**
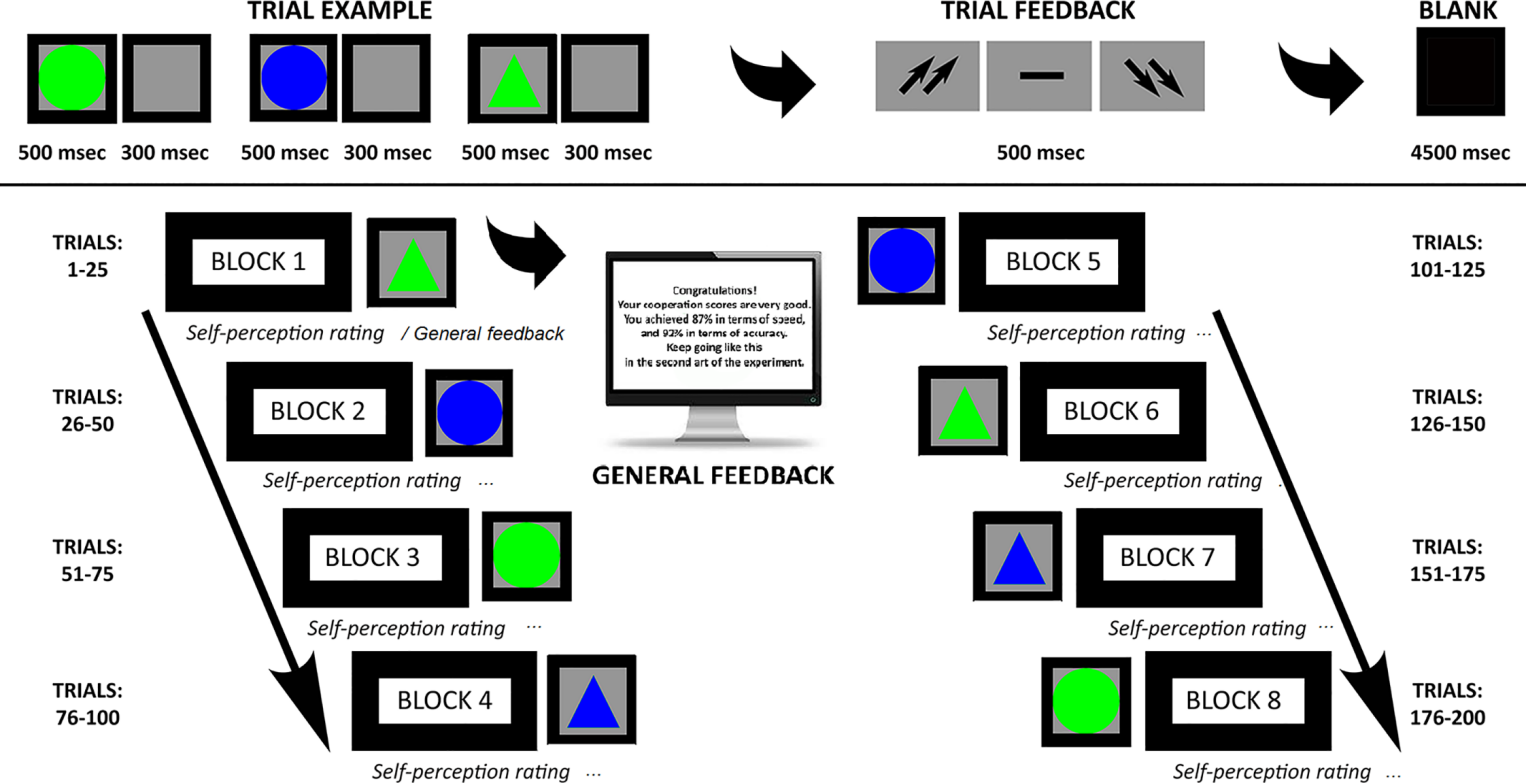
Experimental task. Experimental procedure representing the setting and the experimental tasks.

The task was composed by eight blocks (of 25 trials each) (Fig 1). Thus, participants constantly received a general evaluation of their cooperative performance: both trial feedback (every three stimuli) and general feedback. Both trial feedback and general feedbacks were experimentally manipulated a priori, and after each block, subjects were told by the computer they had good cooperation (“you obtained a good cooperation score, 87% in terms of speed, and 92% in terms of accuracy”) (general feedback) (Fig 1). They were also encouraged to keep up their performance level during the experiment. During the task, after an initial medium performance, subjects were constantly reinforced about their good cooperation by presenting up-arrows in 70% of cases, while the dash or the down-arrows were presented in 30% of cases (trial feedback) (it was preliminarily explained to the subjects that these symbols represent a good or a bad trial feedback on their performance, respectively). In addition, after each block of 25 trials, subjects were required to assess their performance and cognitive efficacy in terms of their ranking position on a seven-point Likert scale (“did you improve your performance?”, from one = most decreased ranking based on how they perceived their performance to seven = most improved ranking based on how they perceived their performance). Specific analysis (ANOVA applied to Likert scale) showed significant increased self-ranking after external feedback across the eight blocks (*F*[1, 25] = 10.45, *p* ≤ .001, η^2^ = .41) with higher self-ranking for each block compared to the previous ones (significant differences *p* ≤ .001 for all comparisons). Participants were strongly engaged in the cooperative context (93% told to be strongly engaged, as reported in a post-experimental questionnaire).

Subjects were also required to self-report their degree of trust of the exact feedback of the performance, which showed high trust (95%), a relevance of the task for social status representation (96%), and the perception of improved ranking position during the task (94%).

### Performance scoring

The RTs (msec) were recorded from the stimulus onset, and ERs were calculated as the total number of incorrect detections out of the total trial for each category. Therefore, higher values represented increased incorrect responses.

### fNIRS

**Fig 2:**
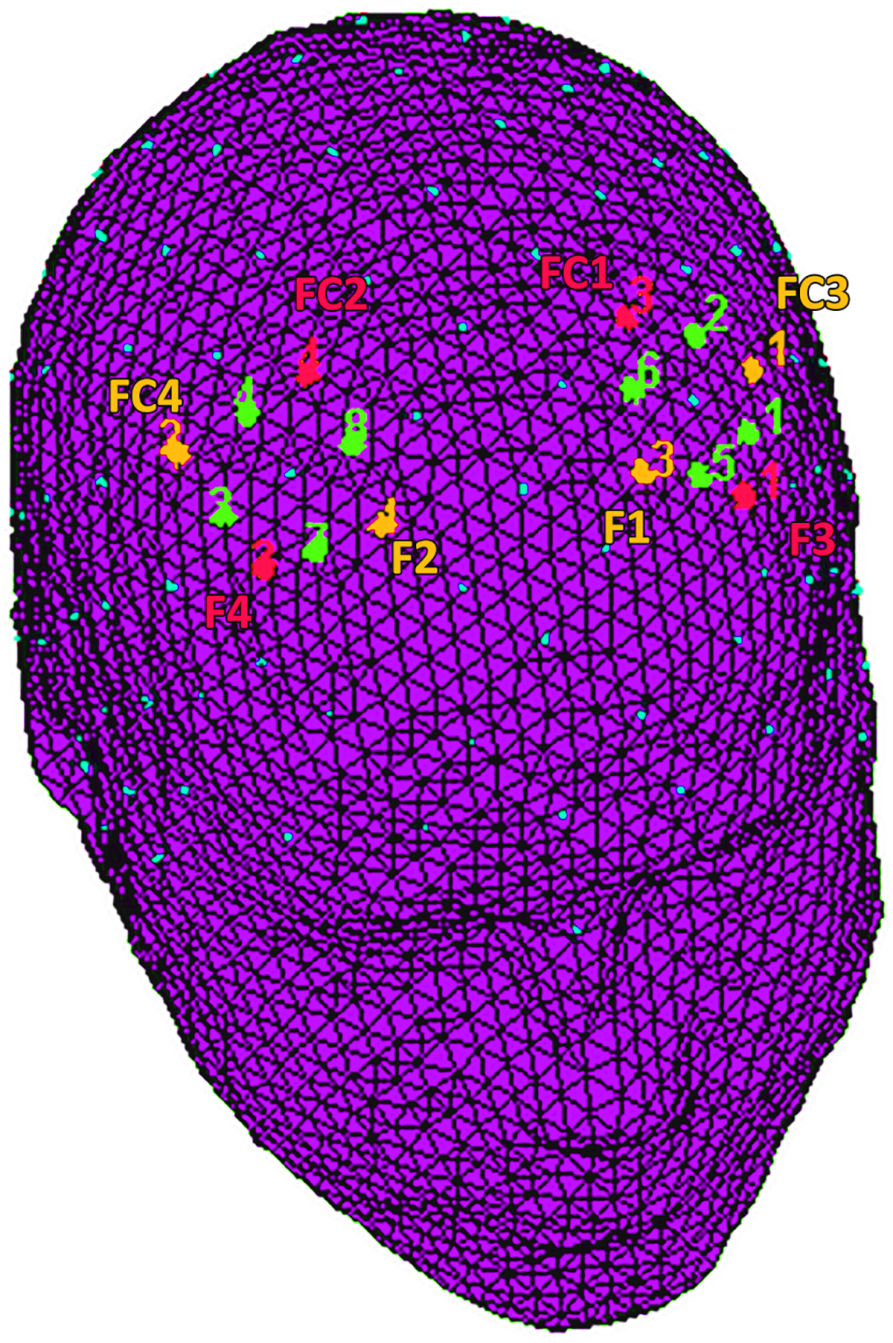
fNIRS montage. The location of NIRS channels. The emitters (orange) were placed on positions FC3-FC4 and F1-F2, while detectors (red) were placed on FC1-FC2 and F3-F4. The resulting channels (green) were as follows: Ch 1 and Ch 3 correspond to the left and right DLPFC. Ch 2 and Ch 4 correspond to the left and right DPMC. Ch 5 and Ch 7 correspond to the left and right FEF. Ch 6 and Ch 8 correspond to the left and right SFG.

fNIRS measurements were conducted with NIRScout System (NIRx Medical Technologies, LLC. Los Angeles, California) using an 8-channel array of optodes (4 light sources/emitters and 4 detectors) covering the frontal and prefrontal area. Emitters were placed on positions (FC3-FC4 and F1-F2), while detectors were placed on FC1-FC2 and F3-F4) (Fig 2). Emitter-detector distance was kept at 30 mm for contiguous optodes and near-infrared light was used at two wavelengths (760 and 850 nm). NIRS optodes were positioned on the subject's head using an NIRS cap according to the international 10/5 system. The following channels were reported: Ch 1 (FC3-F3) and Ch 3 (FC4-F4) correspond to the left and right (respectively) DLPFC (Brodmann Area 9). Ch 2 (FC3-FC1) and Ch 4 (FC4-FC2) correspond to the left and right (respectively) Dorsal Pre-motor Cortex (DPMC, Brodmann Area 6). Ch 5 (F1-F3) and Ch 7 (F2-F4) corresponding to the left and right (respectively) Frontal Eye Fields (FEF, Brodmann Area 8). Ch 6 (F1-FC1) and Ch 8 (F2-FC2) correspond to the left and right (respectively) Superior Frontal Gyrus (SFG, Brodmann Area 6) [27].

Changes in the concentration of oxygenated (O2Hb) and deoxygenated hemoglobin (HHb) were recorded continuously throughout the task with NIRStar Acquisition Software that started from a 120-s resting baseline. Signals obtained from the 8 NIRS channels were acquired with a sampling rate of 6.25 Hz and analyzed and transformed with nirsLAB software (v2014.05; NIRx Medical Technologies LLC, 15Cherry Lane, Glen Head, NY, USA) according to their wavelength and location, which resulted in values for the changes in the concentration of oxy and deoxygenated hemoglobin for each channel, which was scaled in mmol*mm.

The raw O2Hb and HHb data from each channel were digitally bandpass filtered at 0.01–0.3 Hz. Then, the mean concentration of each channel was calculated by averaging data across the trials, starting from the feedback onset for the following 4500 msec. The mean concentration value of 4500 msec before the feedback was used as event-related baseline, where the brain activity is supposed to be at a minimum. According to the mean concentrations in the time series, the effect size in every condition was calculated for each channel and subject. The effect sizes (Cohen’s d) were calculated as the difference of the means of the baseline and trial divided by the standard deviation (sd) of the baseline, which is d = (m1-m2)/s, with m1 and m2 being the mean concentration values during baseline and trial, respectively. The effect sizes (Cohen’s d) were calculated as the difference of the means of the baseline and trial divided by the standard deviation (sd) of the baseline: d = (m1-m2)/s, with m1 and m2 being the mean concentration values during the baseline and trial, respectively, as well as the SD of the baseline. Then, the effect sizes obtained from the 8 channels were averaged to increase the signal-to-noise ratio. Although NIRS raw data were originally relative values and could not be directly averaged across subjects or channels, effect sizes normalized data could be averaged regardless of the unit since the effect size is not affected by differential pathlength factors (DPF) [28–30]. Finally, the event-related responses to stimuli with respect to each baseline signs have been inverted.

### Functional connectivity analysis

Three sets of analyses were performed with respect to behavioral (ERs; RTs) and neurophysiological (fNIRS: O2Hb measures) measures.

First, a repeated measure ANOVAs with the independent factor set as Condition (Cond: pre- vs. post-feedback) was applied to ER, and RTs.

Second, a set of analyses were applied to the neurophysiological level, which consisted of four different steps. First, to obtain intra- and inter-brain connectivity, the partial correlation coefficient Π_*ij*_ was computed to obtain functional connectivity indices. They were obtained by normalizing the inverse of the covariance matrix Γ = Σ^−1^:

$$\Gamma =(\Gamma_{ij})=\Sigma^{-1}:\;\mathrm{inverse}\;\mathrm{of}\;\mathrm{the}\;\mathrm{covariance}\;\mathrm{matrix}$$

$$\Pi_{ij}=-\frac{\Gamma_{ij}}{\sqrt{\Gamma_{ii}\Gamma_{jj}}}:\;\mathrm{partial}\;\mathrm{correlation}\;\mathrm{matrix}$$

This quantifies the relationship between two signals (i, j) independently from each other [31].

Then, we applied ANOVAs to intra- and inter-brain measures. A successive phase included the calculation of a specific ConIndex as the ratio between inter-brain and intra-brain connectivity (Inter_con_/Intra_con_) to directly compare the two connectivity levels. Finally, we applied ANOVA to a ConIndex dependent measure.

For ANOVA, the independent repeated factors were Condition (Cond), Localization (Loc: DLPFC, DPMC, FEF, SFG) and Lateralization (Lat: left vs. right). For all the ANOVA tests, the degrees of freedom were corrected using Greenhouse–Geisser epsilon when appropriate. Post hoc comparisons (contrast analyses) were applied to the data. A Bonferroni test was applied for multiple comparisons. In addition, the normality of the data distribution was preliminary tested (kurtosis and asymmetry tests). The normality assumption of the distribution was supported by these preliminary tests.

Finally, a third step included a correlation analysis to compare behavioral (RTs and ERs) and neurophysiological (intra-brain connectivity; inter-brain connectivity; ConIndex) measures to explore the co-modulation of these different levels with each other.

To exclude a possible learning effect due to pre/post-feedback condition, a preliminary analysis was conducted, comparing distinctly the first set of four intervals (pre-feedback intervals) and the second set of four intervals (post-feedback) for all the dependent measures (RTs, ERs, O2Hb). No significant differences among the four intervals (respectively before and after the feedback) were found. Therefore, we did not include this factor in the remaining analyses.

## Results

### ERs and RTs

**Fig 3:**
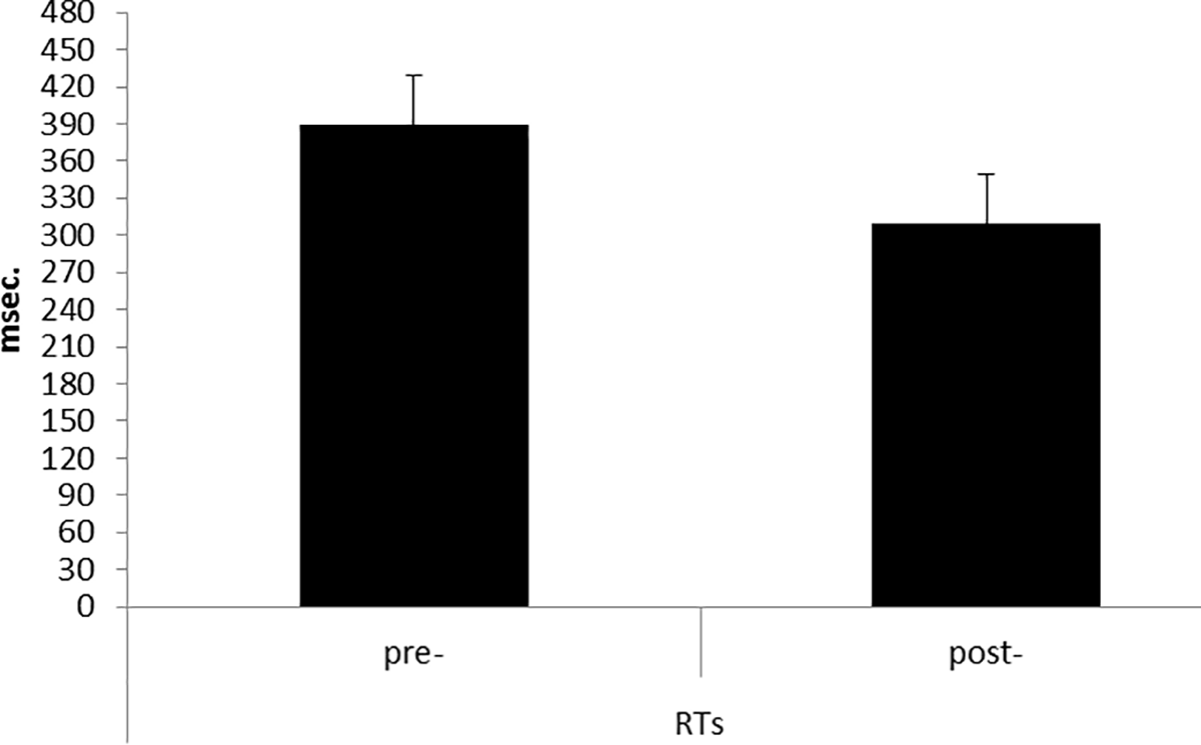
Behavioral results. RTs modulation as a function of Condition (pre vs post). The speed performance was characterized by faster RTs during the post-feedback condition.

For ERs measurement, ANOVA did not show significant effects for Cond (*F*[1, 25] = 1.90, *p* = .12, η^2^ = .17). In contrast, as for RTs, ANOVA indicated a significant main effect for Cond (*F*[1, 25] = 9.05, *p* ≤ .001, η^2^ = .39) with decreased RTs during post-feedback compared to pre-feedback conditions (Fig 3).

### Intra-brain connectivity

**Fig 4:**
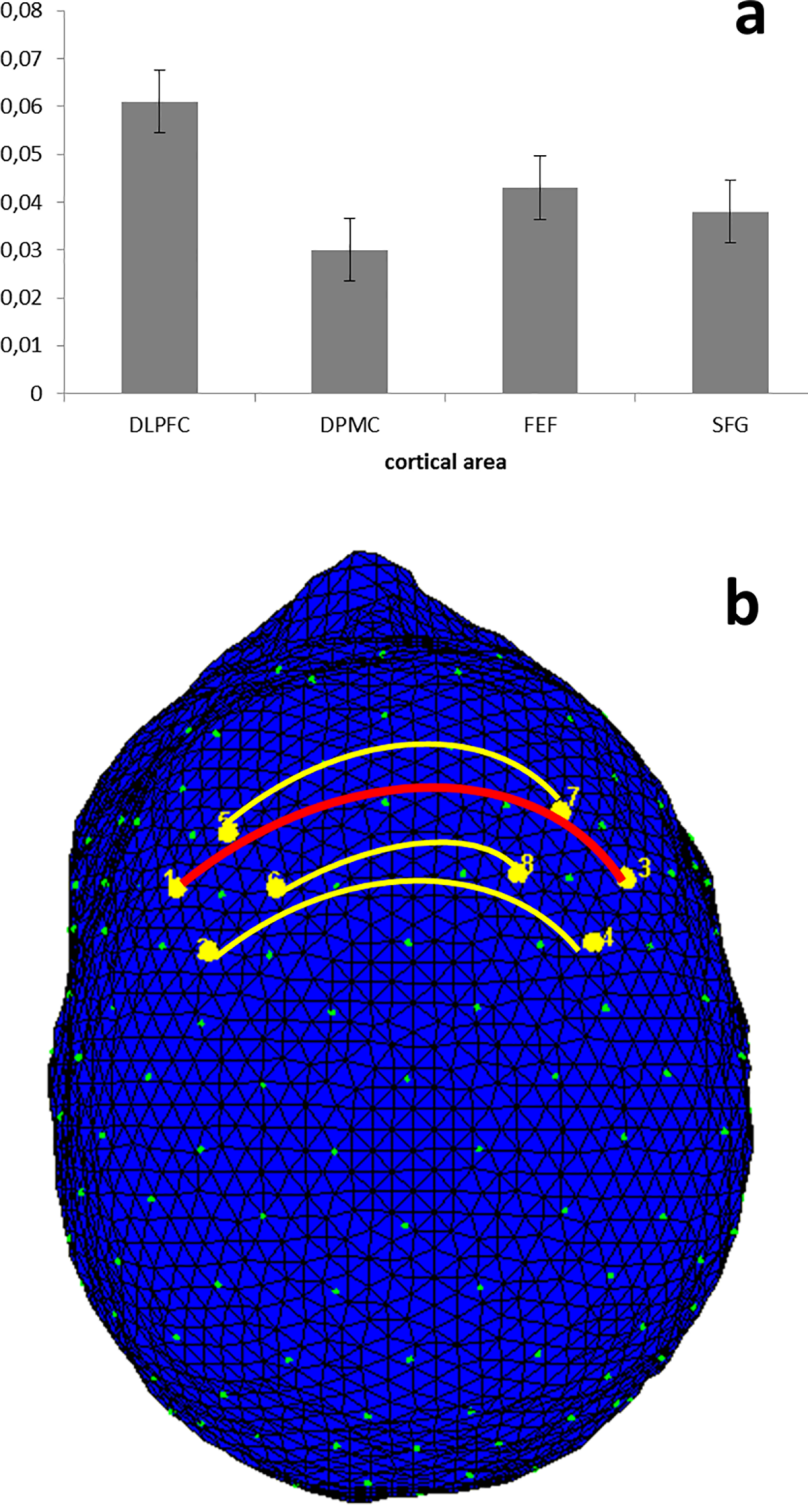
Intra-brain connectivity. Bar graph (a) and intra-brain connectivity map (b) of O2Hb correlations as a function of Localization (averaged for pre- and post-feedback). The results showed that intra-brain connectivity was generally higher in DLPFC than in other areas, independently from the pre- or post-feedback.

The statistical analyses were applied to intra-brain indices for O2Hb and HHb-concentrations. The analysis on HHb did not reveal significant effects, and for this reason we reported only results for O2Hb-values. As shown by ANOVA, the Loc effect was significant (*F*[1, 83] = 11.34, *p* ≤ .001, η^2^ = .40). As revealed by post hoc analysis, intra-brain connectivity was generally higher in DLPFC compared to other areas (compared to DPMC *F*[1, 24] = 7.39, *p* ≤ .001, η^2^ = .35, FEF *F*[1, 24] = 8.13, *p* ≤ .001, η^2^ = .34, SFG *F*[1, 24] = 9.08, *p* ≤ .001, η^2^ = .37) (Fig 4). No other simple or interaction effect was significant.

### Inter-brain connectivity

**Fig 5:**
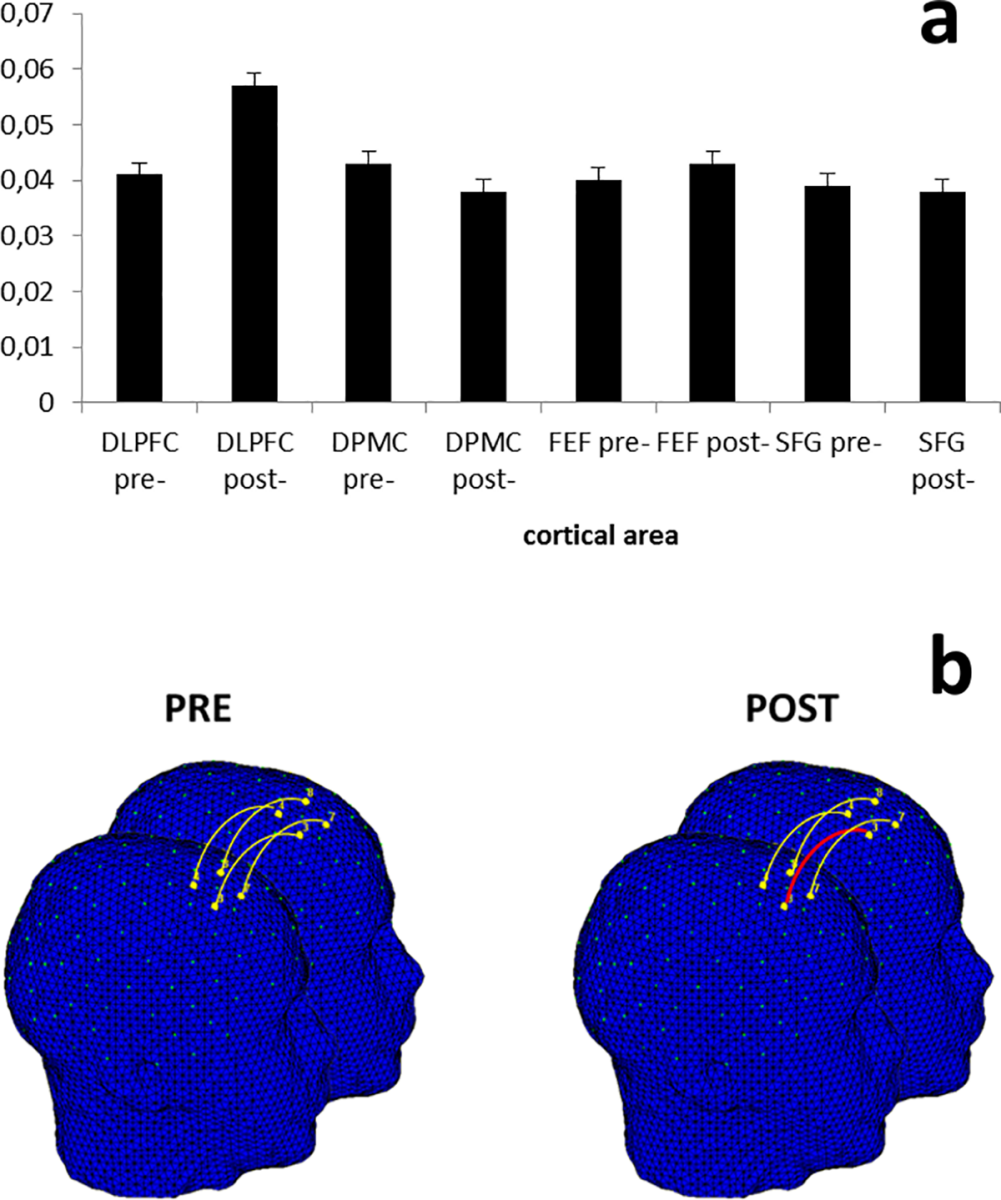
Inter-brain connectivity. Bar graph (a) and inter-brain connectivity map (b) of O2Hb correlations as a function of Condition and Localization. The results showed that inter-brain connectivity increased in the post-feedback stage compared to pre-feedback in DLPFC.

The ANOVA applied to inter-brain indices for the dyads showed a significant Cond main effect (*F*[1, 12] = 10.45, *p* ≤ .001, η^2^ = .33) and a Cond × Localization interaction effect (*F*[1, 36] = 9.67, *p* ≤ .001, η^2^ = .38). Indeed, for the main effect, increased inter-brain connectivity was observed in the post-feedback condition compared to pre-feedback condition. In addition, as revealed by a significant interaction (simple effects), inter-brain connectivity increased in post-feedback compared to pre-feedback in DLPFC (*F*[1, 12] = 10.45, *p* ≤ .001, η^2^ = .41) (Fig 5).

### ConIndex

**Fig 6:**
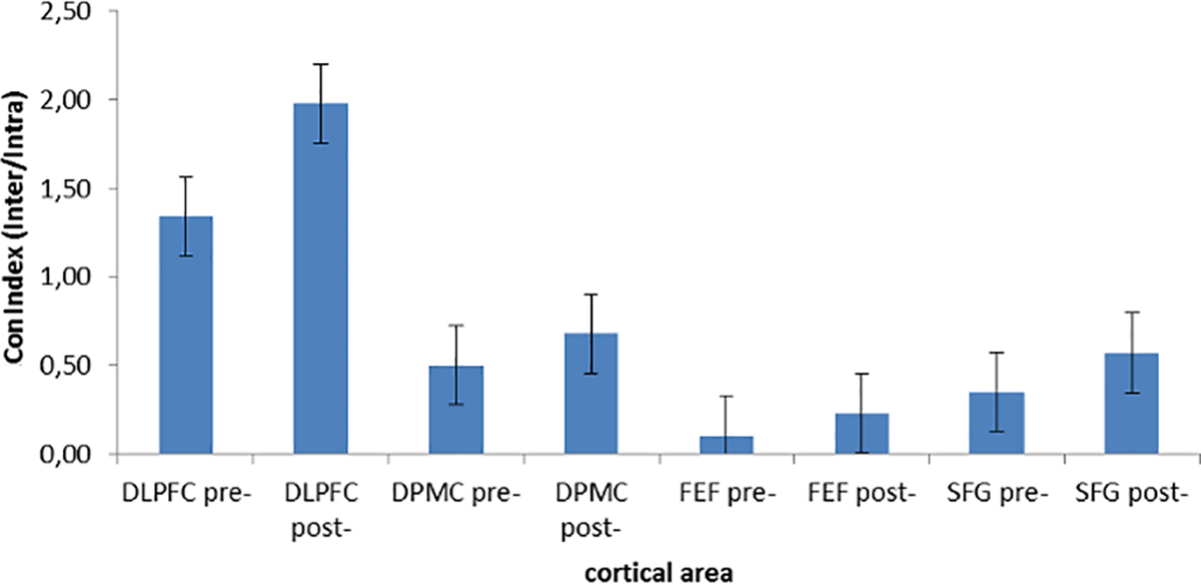
ConIndex results. Increased Index was observed in post-feedback than pre-feedback condition within the DLPFC, thus suggesting a general increased inter-brain connectivity compared to intra-brain connectivity in DLPFC for the post-feedback condition.

The ANOVA applied to ConIndex showed a significant Cond x Localization interaction effect (*F*[1, 36] = 9.67, *p* ≤ .001, η^2^ = .38). Indeed, an increased Index was observed in post-feedback than the pre-feedback condition within the DLPFC. Therefore, as shown by the Index, we can conclude there is a general increase in inter-brain connectivity compared to intra-brain connectivity in DLPFC for the post-feedback condition (Fig 6).

### Correlation analysis

**Fig 7:**
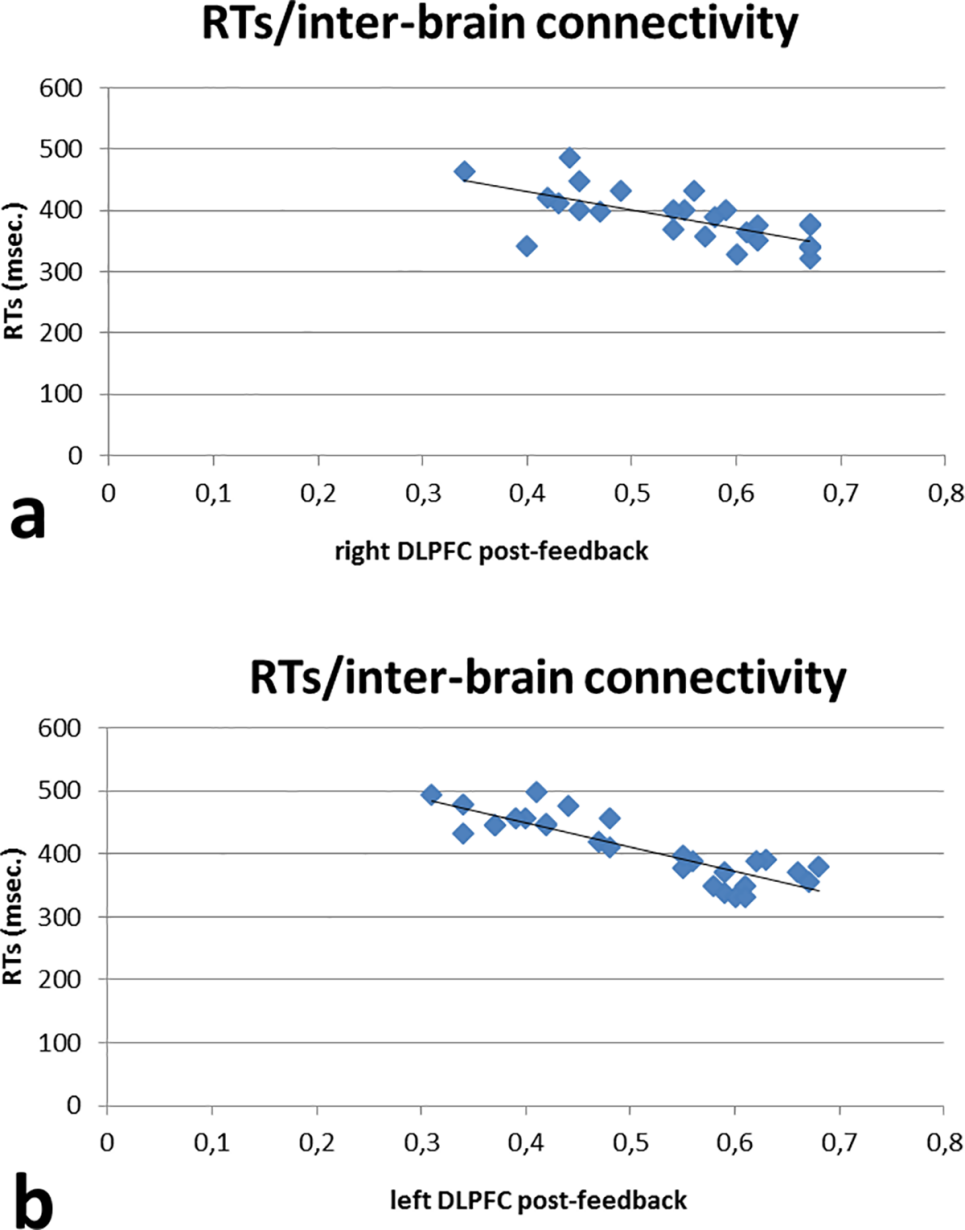
Correlation results. RTs revealed a significant negative correlation with the inter-brain connectivity within right (a) and left (b) DLPFC in post-feedback conditions, such that the increased right and left DLPFC connectivity was related to a reduction of RTs values in post-feedback condition.

Correlation analyses were applied to behavioral (ERs; RTs) and neurophysiological measures (intra- and inter-brain connectivity; ConInd). Corrections for multiple comparisons (Bonferroni corrections) were applied to the analyses. To compare brain connectivity to behavioral performance, RTs and ERs, the initial correlational values obtained within each dyad were calculated and taken as measures for successive correlational analysis. As shown by Pearson correlation coefficients, RTs revealed a significant negative correlation with the inter-brain connectivity within left and right DLPFC in post-feedback condition (respectively r^2^ = —.523, *p* ≤ .001; r^2^ = —.565, *p* ≤ .001): indeed, the increased right and left DLPFC connectivity was related to a reduction of RTs values in the post-feedback condition. No other effect was statistically significant either (Fig 7A and 7B)

## Discussion

The present research study analyzed the behavioral performance and the brain activity (intra- and inter-brain connectivity) during a joint action task where external feedback was used to reinforce good cooperative outcomes.

The modulation of different variables before (pre) and after (post) receiving such feedback has been considered, such as RTs, ERs, and intra- and inter-brain functional connectivity. Additionally, correlations between behavioral and brain activity have been performed.

First, as predicted, the results showed that the external feedback could modulate participants’ responses in both behavioral and neural components. In fact, after the reinforcing feedback, RTs were faster and inter-brain connectivity indices were higher than in pre-feedback condition. According to our hypotheses and to results obtained in previous research, the perception of positive feedbacks, and subsequently of self-efficacy, is able to induce improved behavioral performance in different educational [32,33], sport [34], and work [35] contexts. Such effects were also related to the activation of prefrontal sites, which are usually triggered by an increase in cognitive synergy and brain-to-brain coupling [36]. Therefore, we could speak about a general “gain” due to positive reinforcement. The feedback, in fact, may have generated a strategic joint trend and boosted optimization of the cognitive performance within the dyad.

However, in the present research, we also explored the contribution of specific frontal areas in the different experimental conditions more directly. In detail, we found that the increase in cortical connectivity was mainly localized within the DLPFC for all the neurophysiological measures. This trend is particularly relevant since both intra-brain and inter-brain indices supported the recruitment of a brain area involved in social exchange. In fact, previous research underlined the role of DLPFC not only in perspective and theory of mind [20] but also in the suppression of selfish behavior [21] and commitment in significant relationships [22], even if in inter-brain patterns in the DLPFC have emerged only after social reinforcement. The same results were obtained with ConIndex, which suggests that the extent to which the two indices contribute to such activation is greater in inter-brain correlation. Therefore, the supplementary contribution the inter-brain with respect to intra-brain connectivity was substantial in the second part of the task. Interestingly, intra-brain connectivity **was not affected** by the feedback but only showed an increased pattern of connection between the two DLPFC. This finding suggests that the engagement of DLPFC after social feedback can be referred to as the adoption of joint strategies, while the increased connectivity between homologous DLPFC in intra-brain analysis can suggest the general recruitment of a neural network for the joint task. Previous results already revealed that prefrontal areas are fundamental in social status regulation and joint actions [37–39]. Additionally, using an EEG-based hyperscanning technique, specific DLPFC activation emerged during reciprocal interactions [40].

Moreover, it has been demonstrated that only the “two-players connection” may guarantee an efficient performance, as revealed by the presence of significant correlations between RTs and inter-brain connectivity indices, which confirms a “reinforced trend.” In fact, we may suppose that the external reinforcement could have modulated the effective joint behavior with increased cognitive performance and inter-brain activity by two inter-agents. In other terms, we may suggest that the two levels, behavioral and cortical, were effective in signaling the social effect of the reinforcing feedback. Additionally, they showed similar responsiveness to external conditions that stress the joint significance of inter-subjective actions.

In addition, compared to other previous research, which included both negative feedback on cooperative actions [41] or competitive tasks [42,43], the present study could induce different and specific effects, including improving cognitive performance and the increased inter-brain connectivity (but not the intra-brain connectivity), which specifically represents cooperative and positively reinforced cooperative conditions.

Finally, the significance of the correlation between behavioral performance (RTs reduction) and the inter-brain connectivity, but not between RTs and intra-brain connectivity, may suggest that a brain-to-brain coupling induced by a cooperative task may be directly associated with a significantly improved performance. However, we do not suppose that the brain affects behavior, or vice versa, because it falls short of a “causal model.” We only observed brain and behavioral changes related to the experimental conditions and their possible relations. That is, we do not suppose that inter-brain functional connectivity may directly affect behavior or, in the other direction, that behavioral changes affect the strength of inter-brain connectivity. We can only assume that based on the present social task, two or more brains may function in a more synergic way (as shown by the connectivity values based on correlation) and that, at the same time, the behavioral performance may become more synchronous (more coherent RTs).

As demonstrated by other experiments and the present results, during social interactions, people may significantly affect and shape each other’s behaviors [44] through basic resonance mechanisms. Recent research proposed that during social exchange, such synchronization can actually occur in the form of an alignment of behavior [45,46], posture [47] as well as neurophysiological [3,48] and psychophysiological measures [49–53].

Previous hyperscanning approaches have already highlighted some patterns of behavioral synchronization for their cooperation by EEG [54–57] or functional near-infrared spectroscopy (fNIRS) [6,13,17,58,59].

As shown by previous studies and by our results, the “synergic brain” may support better social interactions with benefits for all the actors involved. For this reason, a consistent and relevant improvement could be observed between the participants, who may benefit from the synergy established between the neuroanatomical networks with a significant gain for the coordination of behavioral activities. In other words, a good synchronization based on a synergic strategy is advantageous for the efficiency of joint behavior, which benefits in turn by the higher coordination between our brains, which learn to “communicate” to support the behavioral level.

Such results are also crucial from a neuroanatomical point of view, since the involvement of prefrontal areas have been associated with social exchange. Thus, after receiving a positive feedback about a dyad’s synchrony, increased connectivity might emerge in areas related to empathy, bonding, and, importantly, in the suppression of self-centered behaviors in favor of a common goal.

The present results could also be explained taking into account the “attentional effect” that was also found to induce a significant increased inter-brain connectivity in the case of a joined task [19]. However, two main considerations should also be reported. In the present study, the absence of a significant intra-brain effect may partially require adjunctive explanations, since a positive feedback (although on a joined-action level) should also have improved the individual attentional mechanism. Additionally, we may suppose that this attentional effect could not have been sufficient to support the enduring and constant increased performance (and brain connectivity) during the entire task, since, as shown by previous research, the attentional effect appears to decline over time. Therefore, the motivational and social reinforcement may have acted as a relevant sustained factor to produce both behavioral and brain effects during the task.

Some limitations should be reported for the present study. First, the limited number of dyads should be increased in future research. Second, to better evaluate the cortical localization and the functional meaning of the brain coupling effect, the posterior areas of the brain should be included. Therefore, in future research, a global analysis on the cortical sites could be applied. Finally, although the analyses permitted us to isolate the contribution of intra and inter-brain connectivity, a more systematic comparison could be made with other experimental conditions consisting, for example, in competitive strategies, cooperation with negative feedback, or a solitary game.
